# Local level health preparedness for adverse weather: A review of Community Risk Registers in England

**DOI:** 10.1016/j.joclim.2024.100403

**Published:** 2024-12-17

**Authors:** Agnes Jung, Richard Kopnyicky, Katya Brooks, Emily Loud, Sharif Ismail, Agostinho Moreira de Sousa, Daniel Blake

**Affiliations:** a Extreme Events and Health Protection, Centre for Climate and Health Security, UK Health Security Agency, 10 South Colonnade, London E14 4PU, UK; b Immunisation and Vaccine Preventable Diseases Division, UK Health Security Agency, 10 South Colonnade, London E14 4PU, UK

**Keywords:** Adverse weather, Risk management, Emergency planning

## Abstract

**Background:**

The increasing frequency of adverse weather events due to climate change poses challenges for emergency planning, response and recovery. While many countries have national plans for preparedness and response to specific hazards, the extent to which these plans influence local health risk management strategies is unclear.

**Methods:**

An assessment of Community Risk Registers (CRRs) published by Local Resilience Forums (LRFs) in England was conducted in 2023. The assessment criteria applied spanned recognition of disaggregated adverse weather risks, through to incorporation of guidance from national agencies within the CRRs.

**Results:**

Of the 33 (out of 38) CRRs reviewed, only half referenced adverse weather risks, and around half referenced relevant national and local guidance to support preparedness and response to minimise potential health impacts. Only two CRRs met all assessment criteria (i.e., the referencing of adverse weather risks, as well as national, local and UK Health Security Agency (UKHSA) specific public health guidance on adverse weather risks).

**Conclusions:**

There is a need to support strengthened inclusion of national evidence and guidance into local risk assessments and the translation of these into relevant documents to raise public awareness of the health impacts from adverse weather.

## 1 Introduction

Risks to human health from adverse weather in the UK are substantial and increasing, in part due to climate change [[1], [2], [3]]. Health risks from extreme heat, flooding and drought, are projected to increase [4], and cold weather is projected to continue to account for a substantial burden of death and disease [5].

The UK Health Security Agency (UKHSA) – the government agency responsible for public health protection across England – published an Adverse Weather and Health Plan (AWHP) in 2023 setting out recommendations for preparedness, alerting and response to reduce the health impacts of adverse weather (including heatwaves, cold events, floods and drought) [6]. However, the extent to which adverse weather risks are assessed and prepared for in local level emergency planning is variable. Global evidence points to a disconnect between national guidance and local level implementation in many settings [7,8].

The Civil Contingencies Act 2004 (CCA 2004) and accompanying non-legislative measures, provide the framework for civil protection in the UK [9]. The Act divides local responders into two categories with different sets of duties. Responders come together in Local Resilience Forums (LRFs) with voluntary organisations, utility service companies and businesses and form local multi-agency partnerships who work to identify and assess potential local risks, produce generic and specific multi-agency emergency plans, and respond to incidents in a coordinated manner [10].

Local risks in the UK are theoretically documented and made public in each LRFs’ Community Risk Register (CRR). CRRs focus on local risks with the highest likelihood and the greatest potential for risks to life, and disruption to services and communities in their locality. CRR documents are typically based on information from the National Risk Register for the UK [11] and inform local emergency planning, preparedness activities and associated public awareness.

We assessed how LRFs in England address adverse and extreme weather risks within CRRs as a key part of the UK's adaptation to global climate change. CRRs were reviewed for any referencing of specific adverse weather hazards described in the 2023 UKHSA's AWHP (i.e., heat, cold, flooding and drought) [6] and whether national and local guidance for related adverse weather health risks was incorporated into CRRs.

## 2 Methods

CRRs published by LRFs in England were assessed using a set of criteria between July and October 2023. Criteria were pre-established through expert elicitation internally within UKHSA (involving staff with backgrounds and professional experience in risk management and public health) as a technique to audit key LRF adverse weather responsibilities. CRRs were downloaded from publicly accessible websites [10]. LRFs were also directly contacted to obtain CRRs.

CRR content was assessed using the four criteria outlined in Table 1.

**Table 1:** Criteria used to assess Community Risk Registers in England and importance of each criterion in representing Local Resilience Forum responsibilities related to adverse weather.

Criteria	Importance
1. Whether adverse weather risks were disaggregated by the four hazard types outlined in the national UKHSA Adverse Weather and Health Plan (i.e., heat, cold, flooding and drought separately) [6] rather than consideration as a single collated group of hazards.	Ensures targeted risk management.
2. Presence of webpage links and references to national guidance documents and information provided by all national organisations on adverse weather and health risks, preparedness and response.	Enables Local Resilience Forums to access critical information for preparedness and response.
3. Presence of reference to local guidance or services relevant to adverse weather and health risks, preparedness and response.	Indicates that national recommendations are adapted to local contexts.
4. Presence of webpage links and references to UK Health Security Agency public health guidance on adverse weather and health risks, preparedness and response.	Highlights the UK Health Security Agency's role in issuing Weather-Health Alerts and providing actionable tools to safeguard public health.

Each criterion was assessed as “met” if any evidence was documented against each, and “not met” if no evidence was documented. An aggregate assessment across all four criteria was then generated: if all criteria were met, the CRR was classified as “criteria fully met”. If none were met, then it was assessed as “criteria not met”. All others were classified as “criteria partially met”.

## 3 Results

CRRs were obtained for 33 of 38 LRFs across England. Of the 33 CRRs reviewed, only two met all four assessment criteria (6%). Eight CRRs (24%) didn't meet any assessment criteria. The remaining 23 met some but not all the criteria.

Incorporation of adverse weather hazards across the CRRs was highly variable. Two (6%) CRRs made no reference to adverse weather at all (either in aggregate or for specific adverse weather hazards). Around half (52%) of the assessed CRRs distinguished between the four types of adverse weather hazards (i.e., heat, cold, flooding and drought) outlined in the AWHP.

**Fig. 1:**
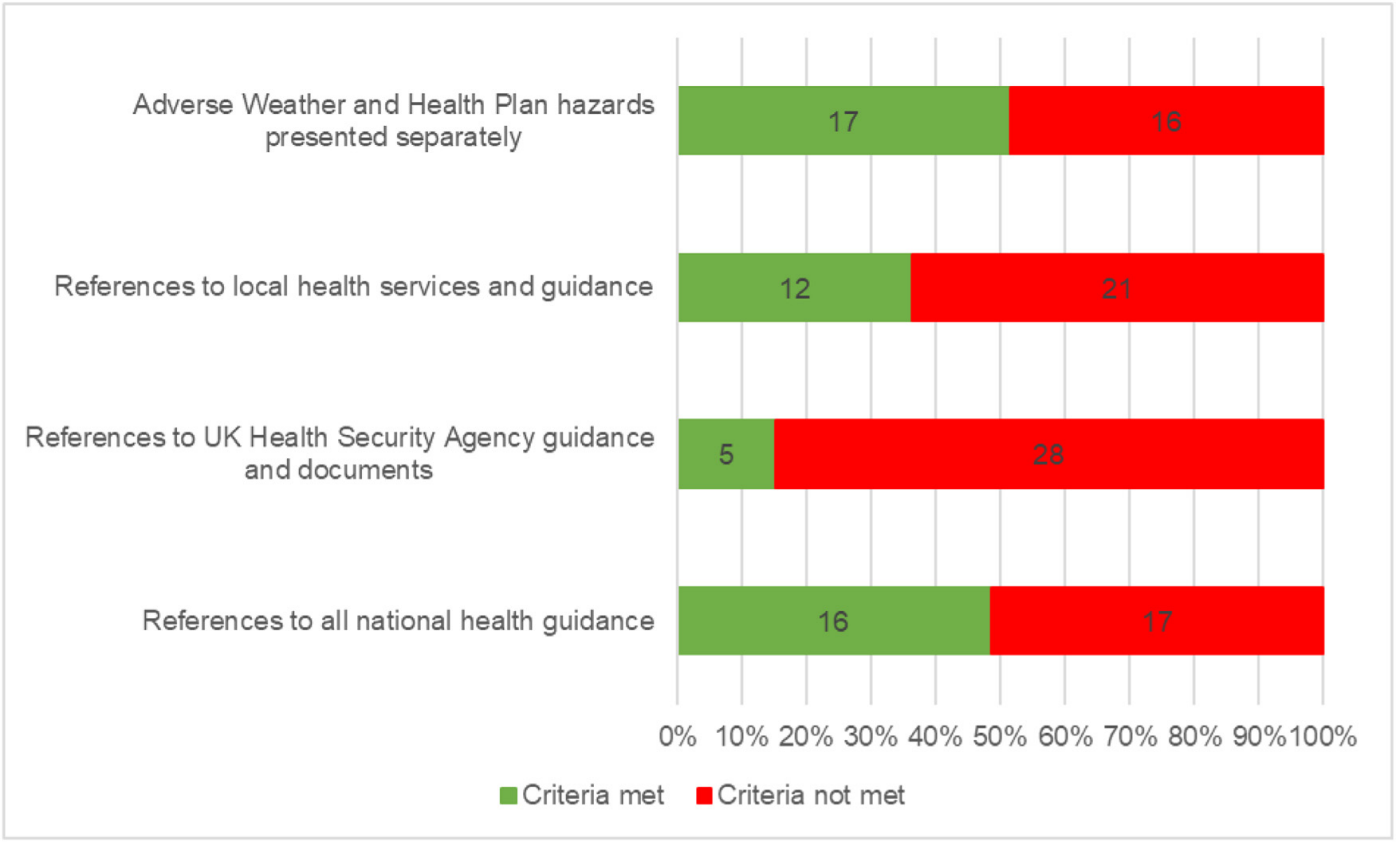
Proportion of Community Risk Registers in England included in the analysis meeting each of the four individual assessment criteria used in this study.

The extent to which wider national guidance and advice was considered in CRRs also varied. Around half (48%) of the documents included references and links to health guidance from national bodies besides the UKHSA, such as NHS England. Only five (15%) of CRRs included links or references to published UKHSA guidance (Fig. 1).

## 4 Discussion

This analysis considered the extent to which adverse weather hazards are incorporated into local-level CRRs in England, and the extent to which these registers incorporate recognised national and local level guidance on reducing health risks. Findings reported here emphasise the need to further build understanding and appreciation of adverse weather risks to health among actors involved in local preparedness and response across England.

While CRRs are intended to reflect appraisals of local risks, around half of CRRs examined considered all adverse weather hazards as a single category. This is despite differences in risks to health dependent on the type of hazard [12]. Incorporation of relevant local webpage links and national guidance from various organisations including the UKHSA was variable.

These findings mirror other evidence from the UK and internationally that much of current planning for different adverse weather hazards is oriented to national level actors, and that there is considerable scope for strengthening local mechanisms to support this [7,8]. Strengthening local risk appraisal and preparedness is crucial for ensuring effective responses and the long-term resilience of local systems and communities to the health impacts of adverse weather [13].

Limitations to this analysis are that:

• The assessment was carried out before the most recent update to the National Risk Register for the UK, published in 2023. Furthermore, UKHSA's AWHP was released in 2023 (soon before the study assessment) and has since been updated. Results should therefore be viewed as a single snapshot in time, with updates to CRRs possibly prompted by the updates of such guidance, albeit with a lag in time. However, we note that documents with similar content existed before 2023, including earlier versions of the National Risk Register, AWHP and Public Health England's (whose functions were later absorbed by UKHSA) hot and cold weather plans [e.g., [14], [15], [16]].
• The absence of references or links to external sources within CRRs does not definitively establish that these were not considered by LRFs when compiling these documents.
• It was not possible to review all CRRs for LRFs in England because some were not publicly available at the time of the study and could not be obtained from representatives.

Future work should seek to improve the understanding of approaches taken by LRFs for local risk assessments to identify and assess specific adverse weather hazards and risks within CRRs. Public awareness of health impacts from adverse weather could be improved by ensuring that all CRRs are always publicly available. An improved understanding is also needed of mechanisms for facilitation and prevention of uptake of national and local guidance on preparedness for adverse weather risks. These findings will inform future activities under the AWHP to bolster health protection and resilience of communities to adverse weather events in England.

## 5 Conclusion

The study of CRRs in England reveals inconsistencies in the representation of local risk assessments and the sharing of related information on the health impacts of adverse weather hazards. Incorporation of adverse weather risks across the CRRs was highly variable. Only two (6%) of the assessed CRR's fully met all assessment criteria. Approximately half (52%) addressed risks from distinct adverse weather hazards (i.e. flooding, cold weather, extreme heat). Approximately half (48%) addressed adverse weather events through incorporating national guidance. Two (6%) made no reference to adverse weather at all.

The findings emphasise a critical need for improved inclusion and translation of evidence and guidance into CRRs to inform local health risk appraisals and preparedness measures. Future efforts should focus on addressing each adverse weather risk separately and understanding and overcoming barriers to incorporation of national and local guidance. This will enhance community response strategies and resilience.

## Funding

This study is funded by the National Institute for Health and Care Research (NIHR) Health Protection Research Unit (HPRU) in Environmental Change and Health (NIHR 200909), a partnership between UK Health Security Agency and between UKHSA and the London School of Hygiene and Tropical Medicine (LSHTM), in collaboration with University College London and the Met Office. The views expressed are those of the author(s) and not necessarily those of the NIHR, UK Health Security Agency, London School of Hygiene and Tropical Medicine, University College London, the Met Office or the Department of Health and Social Care.

## CRediT authorship contribution statement

**Agnes Jung:** Writing – review & editing, Writing – original draft, Supervision, Methodology, Investigation, Formal analysis, Conceptualization. **Richard Kopnyicky:** Writing – review & editing, Methodology, Investigation, Conceptualization. **Katya Brooks:** Writing – original draft, Methodology, Investigation, Conceptualization. **Emily Loud:** Writing – review & editing, Writing – original draft, Methodology, Investigation, Data curation, Conceptualization. **Sharif Ismail:** Writing – review & editing, Writing – original draft, Methodology, Investigation, Conceptualization. **Agostinho Moreira de Sousa:** Writing – original draft, Conceptualization. **Daniel Blake:** Writing – review & editing, Writing – original draft, Investigation.

## Declaration of competing interest

The authors declare that they have no known competing financial interests or personal relationships that could have appeared to influence the work reported in this paper.
